# 

**Published:** 1896-12

**Authors:** 


					CONTENTS.
The Evils of Marriage and Preg-
nancy in Women who do not
possess Sound Pelvic Organs.
?By A. E. Aust Lawrence, M.D.,
C.M. Aberd
289
A Case of Double Spasmodic Torti-
collis: Excision of both Spinal
Accessory Nerves.
By R. Shingle-
ton Smith, M.D., B.Sc. Lond.,
F.R.C.P. Lond
297
The Diagnosis and Treatment of
ijumbago.
By F. H. Edgeworth,
M.B., B.A. Cantab., B.Sc. Lond. ...
30$
wotes on Self-Poisoning and it?
Treatment, with Special Refer-
ence to some Physical Methods.
tfy C. J. Whitby, B.A., M.D. Cantab.
307
Progress of the Medical Sciences:
Medicine.
By J. Michell Clarke, I
M.D.,andG. A. Bannatyne, m.u.
317
Surgery.
By James Swain, M.D....
330
Laryngology.
By Barclay J. Baron,
M.B.
Otology.
By W. H. Harsant
341
Reviews of Books
313
Notes on Preparations for the Sick
368
Meetings of Societies
370
The Library of the Bristol Medico-
Chirurgical Society 373
Local Medical Notes
378
Scraps
380

				

